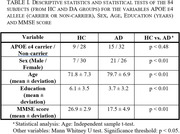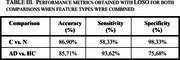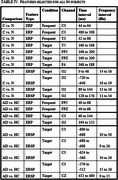# Classification of Apolipoprotein E ϵ4 Allele Carriers in Alzheimer Disease Patients and Healthy Controls using Event‐Related Potentials and Event‐Related Spectral Perturbation

**DOI:** 10.1002/alz.084919

**Published:** 2025-01-09

**Authors:** Kauê O. Frassão, Sandro M. S. Filho, Renata Valle Pedroso, Carla Nascimento, Henrique Pott Junior, Marcia Regina Cominetti, Francisco J. Fraga

**Affiliations:** ^1^ Federal University of ABC (UFABC), Santo André, SP Brazil; ^2^ Instituto de Neurociencias de Alicante, Universidad Miguel Hernández‐CSIC, Sant Joan d'Alacant Spain; ^3^ Federal University of São Carlos, São Carlos, SP Brazil; ^4^ Federal University of São Carlos, São Carlos Brazil; ^5^ Federal University of Sao Carlos, Sao Carlos, SP Brazil; ^6^ Federal University of ABC, Santo André, SP Brazil

## Abstract

**Background:**

Understanding the genetic etiology of Alzheimer's disease (AD) has been a major focus of research in neurodegenerative diseases. Amid the three common allelic variants of the apolipoprotein E (APOE) gene in humans, called APOE ε2, ε3 and ε4, the ε4 allele is the most common genetic risk factor for late‐onset AD, being found in 20% of the world population.

**Method:**

We used Event‐Related Potentials (ERP) and Event‐Related Spectral Perturbation (ERSP) as features for classification of apolipoprotein E ϵ4 (APOE ε4) allele carriers in AD patients and healthy controls. The study participants were 37 healthy older adults (9 APOE ε4 carriers) and 47 AD patients (15 APOE ε4 carriers), which underwent an auditory oddball task (with Target and Frequent trials) using an electroencephalographic (EEG) equipment with 21 channels. A leave‐one‐subject‐out (LOSO) cross‐validation approach was used to perform feature selection and classification with Support Vector Machine (SVM) classifiers.

**Result:**

After feature extraction and selection, we achieved a classification accuracy of 86,90% in the APOE ε4 carriers versus non‐carriers comparison (regardless diagnosis) and 85,71% in the Alzheimer disease patients versus healthy controls comparison (regardless APOE ε4 status). When combining the results of all participants we reached a global accuracy of 73,81% in the four‐class classification (Alzheimer disease patients carriers and non‐carriers, healthy elderly carriers and non‐carriers).

**Conclusion:**

We hope our work could help clinicians to make a more accurate and earlier AD diagnosis, considering the presence of the APOE ε4 allele.